# 

**Published:** 1916-07-15

**Authors:** 


### CONTENTS.

PAGE

**The Twilight Sleep Propaganda: A Danger to the Race** ... ... ... ... ... ... ... 359

**The Public and the General Practitioner** ... 360

**Hospital and Institutional News** ... ... ... 361

Visit of Their Majesties to the South African Hospital—Opening of the New Chelsea Hospital by the Queen—Queen Alexandra and the Police—The Need of Doctors for the Army—A Minister on the Voluntary System—The United States Army Medical Service—Edinburgh University and Women Doctors—The Limitation of Panel Lists—Charing Cross Hospital Thrown Open to Women Students—Home Truths at Aberdeen—The Chaplain is Worthy of His Salary—A Roman Catholic Sanatorium—Suicide of a Swansea Hospital Patient—Infant Hygiene in Wales—The Scottish Women's Organisation—Intoxicants for Military Patients—Red Cross County Histories—Manchester Hospitals and the Battle of the Somme—Unusual Action against a Nursing Home—The Surbiton Red Cross Hospital Secretaryship—The Generosity of Lothian Miners—Clearing Off Debt at Mansfield—The Extensions at Wigan—Surgical Tuberculosis at Newport—Public Libraries and Tuberculosis Infection—The Pay of Poor-Law Doctors Serving at the Front—Margarine *versus* Butter—Guardians' Subscriptions to Hospitals—The July *Gazettes*—The Latest War Hospital *Gazette*—Street Collections for Hospitals—Herb Gardens in Public Parks—This Week's Drug Market.

PAGE

**The Institutional Treatment of Consumption** 367

VI.—Records and Classification of Patients.

**A Medical Causerie** ... ... ... ... ... 369

**Epidemic Jaundice in the Mediterranean Expeditionary Force** ... ... ... ... 370

Research under Difficulties.

**National Insurance Act Developments** ... ... 371

The Interim Report of the Committee of Inquiry.

**The Matrons' and Sisters' Department** ... 373

The Education of the Probationer.

Round the Hospitals.

The College of Nursing—A Matron Fifty Years Ago—A Soldier's Tribute—Thirty Years of Service—Miss H. F. Hopkins—Nurses Wanted for War Work.

**Patriotism and Nurse-training Schools** ... 375

VII.—Training Schools South and West.

**Parliament and the Profession** ... ... ... 376

**The Editor's Letter-Box** ... ... ... ... 377

New South London Hospital for Women: The Operation Unit.

**Hospital Results in 1915** ... ... ... ... 377

**What's What Column** ... ... ... ... 378

**Passing Events and Topics** ... ... ... ... 378

**Gifts to Hospitals** ... ... ... ... ... 378

**The Book Market** ... ... ... ... ... 378

**The Institutional Worker** ... .. (Suppl.) 1

Should Hospital "Letters" be Issued?—Answers to the June Question.

